# Genetic Nature of Elemental Contents in Wheat Grains and Its Genomic Prediction: Toward the Effective Use of Wheat Landraces from Afghanistan

**DOI:** 10.1371/journal.pone.0169416

**Published:** 2017-01-10

**Authors:** Alagu Manickavelu, Tomohiro Hattori, Shuhei Yamaoka, Kazusa Yoshimura, Youichi Kondou, Akio Onogi, Minami Matsui, Hiroyoshi Iwata, Tomohiro Ban

**Affiliations:** 1 Plant Genetic Resources Division, Kihara Institute for Biological Research, Yokohama City University, Yokohama, Kanagawa, Japan; 2 Department of Genomic Science, Central University of Kerala, Riverside Transit Campus, Kerala, India; 3 Department of Agricultural and Environmental Biology, Graduate School of Agricultural and Life Sciences, The University of Tokyo, Tokyo, Japan; 4 College of Science and Engineering, Kanto Gakuin University, Yokohama, Kanagawa, Japan; 5 Centre for Sustainable Resource Sciences, Yokohama, Kanagawa, Japan; Institute of Genetics and Developmental Biology Chinese Academy of Sciences, CHINA

## Abstract

Profiling elemental contents in wheat grains and clarifying the underlying genetic systems are important for the breeding of biofortified crops. Our objective was to evaluate the genetic potential of 269 Afghan wheat landraces for increasing elemental contents in wheat cultivars. The contents of three major (Mg, K, and P) and three minor (Mn, Fe, and Zn) elements in wheat grains were measured by energy dispersive X-ray fluorescence spectrometry. Large variations in elemental contents were observed among landraces. Marker-based heritability estimates were low to moderate, suggesting that the elemental contents are complex quantitative traits. Genetic correlations between two locations (Japan and Afghanistan) and among the six elements were estimated using a multi-response Bayesian linear mixed model. Low-to-moderate genetic correlations were observed among major elements and among minor elements respectively, but not between major and minor elements. A single-response genome-wide association study detected only one significant marker, which was associated with Zn, suggesting it will be difficult to increase the elemental contents of wheat by conventional marker-assisted selection. Genomic predictions for major elemental contents were moderately or highly accurate, whereas those for minor elements were mostly low or moderate. Our results indicate genomic selection may be useful for the genetic improvement of elemental contents in wheat.

## Introduction

Elements, along with nucleic acids, proteins, and metabolites, are essential building blocks of cells, and are involved in almost every process in living organisms [1]. Ionome is defined as “the mineral nutrient and trace element composition of an organism, representing the inorganic component of cellular and organismal systems” [2]. For plants, which take up all elements except carbon and oxygen from the soil, regulating the uptake and distribution of elements from the local soil environment is crucial for survival. For humans, a deficiency of essential elements leads to malnutrition, especially in pregnant women and children below the age of five who suffer from severe acute malnutrition. According to a World Health Organization report, in 2012, 162 and 99 million children are stunted and underweight, respectively, mainly because of insufficient intake of essential nutrients [3]. Cereal-based foods represent the largest proportion of the daily diet in countries with a high incidence of micronutrient deficiencies. The HarvestPlus initiative of the CGIAR consortium is dedicated to alleviating nutrient deficiencies by biofortifying staple food crops with essential minerals and vitamins. This approach is considered to be one of the most economically efficient solutions to human micronutrient deficiency [4–8].

Of the three major staple crops, wheat is the primary source of protein in developing countries, with 2.5 billion consumers in 89 countries worldwide (www.wheat.org). To the best of our knowledge, biofortification efforts involving wheat have been limited, with only one major program that focused on Zn (www.harvestplus.org). Dietary supplements and agronomic practices involving the use of Fe- and Zn-containing fertilizers can help address the nutrient deficiency problem. However, ensuring sufficient nutrient uptake through food is a more sustainable solution. Identifying germplasm with varying elemental contents, and using this variation to breed new cultivars represents a viable solution that takes advantage of the genetic diversity of wheat.

It may be possible to identify unique variations in a characteristic, such as elemental content, using landraces that have evolved to be able to grow under low-input conditions. Landraces originated through agricultural and horticultural practices over the past 10,000 years. They have become highly adapted to diverse environmental conditions, and they have been grown for several millennia in crop centers of origin. Wheat landraces from Afghanistan collected by Dr. Hitoshi Kihara and his colleagues from 1950 to 1970 are an untapped genetic resource that may consist of wheat varieties with an ideal genotype for breeding new cultivars with increased elemental contents. Previous studies revealed considerable genetic diversity in this germplasm [9–11].

Recent technological advances involving molecular markers and high-throughput systems may be useful for the genotypic characterization of many plants [12]. They are well-suited for genomics research, including genome-wide association study (GWAS; [13]) and genomic selection (GS; [14]), for which several markers are required. Genome-wide association studies may identify genomic regions responsible for phenotypic variations in a population at finer resolutions than conventional bi-parental mapping. Additionally, an advantage of GWAS is that it uses the genetic diversity in a germplasm collection without the need for any crossing experiments. Alternatively, GS is a method for predicting genomic breeding values using molecular markers covering the whole genome [14–15]. The use of GS is becoming increasingly popular for plant [16–19] and animal [15, 20] breeding because of recent advances in high-throughput marker technologies and decreases in the cost of genotyping. Low-cost genotyping and rapid generation advancement will greatly reduce the genetic gain per unit cost and time in GS breeding [17]. Genome-wide markers are also useful for estimating the genetic variance and covariance harbored by a population [21], and for estimating heritability and genetic correlations between traits and environments based on the estimated variance and covariance [22].

Ionomics has been extensively used to study model plant species, such as *Arabidopsis thaliana*, to determine the basic functions of various ions or elements (www.ionomicshub.org). In wheat, a few studies have reported the occurrence of genetic variations influencing Fe and Zn contents [23–25]. Recently developed high-throughput techniques, such as inductively coupled plasma and bench-top energy-dispersive X-ray fluorescence spectrometry, have been widely used to study nutrient density. Although the techniques enable the assessment of multiple elements in several samples, the genetic nature (e.g., heritability and genetic correlations among traits and between environments) of elemental contents and the association between phenotypes and genome-wide markers have generally not been studied in detail in crop species [26]. Moreover, because there have been few studies that evaluated the accuracy of genomic predictions for elemental contents [27], further research is necessary to assess the potential of GS for elemental contents.

In the present study, we assessed the variation in elemental contents in wheat seeds using 269 Afghan landraces. We also characterized the genetic nature of elemental compositions by estimating the heritability of elemental contents and the genetic correlations between environments and elements. Additionally, we completed genomic predictions based on 8,465 genome-wide markers and multiple model-building methods, and evaluated the accuracy of the predictions through cross-validations to clarify the potential of GS. The model-building methods were linear and nonlinear to enable the prediction of the genetic mechanisms regulating elemental contents as suggested by Onogi et al. (2015) [28]. We also completed a GWAS to detect major loci controlling elemental contents. We discuss the potential utility of Afghan wheat landraces as resources for future breeding efforts focused on biofortification.

## Materials and Methods

### Plant materials and sampling

We used the whole set of Kihara Afghan wheat landraces (KAWLR), which were registered in the National Bio-Resources Project in Japan, along with 10 Afghan improved wheat varieties as checks to evaluate the phenotypic variations in elemental contents and the underlying genetic basis. Plant materials were grown in an experimental field at the Kihara Institute for Biological Research in Totsuka, Yokohama, Japan using normal field management practices (i.e., 200 kg/ha fertilizer; N:P:K = 8:8:8). Healthy seeds were sown in trays and transplanted to the field before winter in November 2011. For elemental analyses, we chose 267 landraces and seven check varieties that yielded normal grains under the environmental conditions in Japan (Online Resource 1). Border plants were excluded, and grains were harvested from the remaining plants and pooled together for each plot. Twelve grains were selected randomly from the pooled grains for elemental analysis. A subset of landraces was also grown in Afghanistan (2013–2014) using normal field management practices (200 kg/ha diammonium phosphate; N:P = 18:46). Wheat grains were collected and pooled as described above. In Afghanistan, 207 landraces and the same seven check varieties were analyzed (S1 Table). Out of 269 landraces, 205 were evaluated in both Japan and Afghanistan along with the seven check varieties.

### Elemental analysis

An EDX-720/800 HS energy dispersive X-ray fluorescence (EDXRF) spectrometer (Shimadzu, Kyoto, Japan) was used for elemental analysis. Although it enables the measurement of elements in a non-destructive way, we used a destructive method involving a hand-operated pressing tool (Shimadzu) to avoid measuring elements present only in the seed coat and/or aleurone layer. Wheat grains were dried by incubating at 65°C overnight. Four grains were wrapped in weighing paper and pre-crushed with a hammer. The crushed grains were placed between two TC-604 tungsten beads (6.0-mm diameter) (Bio Medical Science) in a tube (master tube hard, MT020-01H; Bio Medical Science) and homogenized for 4 min using a Shaker Master Auto shaker (Bio Medical Science). The powder collected from each tube was placed in a plastic ring (external diameter: 8 mm; internal diameter: 6 mm; and height: 3 mm) without any chemical treatments and pressed at 20–32 MPa to produce a tablet (external diameter: 10 mm; internal diameter: 8 mm; and height: 1 mm). Three tablets per sample were prepared for elemental content measurements. Three major nutrients (Mg, K, and P) and three minor nutrients (Mn, Fe, and Zn) were analyzed. Details on the methodology used are given in Kondou et al [29]. A portion of the resulting elemental concentration data (P, K, Mg and Fe in Japan) have already been reported by Kondou et al [29].

### Genotyping

Total genomic DNA was extracted from 5-week-old leaves of the 269 accessions and seven check varieties using the modified extraction protocol of the DNeasy Plant Mini Kit (Qiagen). Genotyping was completed by Diversity Arrays Technology Pty. Ltd, Yarralumla, Australia. Details regarding genotyping and chromosomal mapping were as described by Manickavelu et al. (2014) [9]. In the present study, DArT markers [30–31] were used in addition to single nucleotide polymorphisms (SNPs). In total, 39,856 markers were used for preliminary analyses. Markers lacking locus information were excluded from the following analysis, which included 8,465 markers. Missing genotypes were imputed using Beagle ver. 3.3.2 [32]. The proportion of missing genotypes in the data of 8,465 markers was 5.6%.

### Data analysis

#### Marker-based heritability and genetic correlation

We estimated heritability and genetic correlations between environments and among elements based on genetic relationships calculated from genome-wide marker polymorphisms.

We estimated the marker-based heritability using the R package “MCMCglmm” [21] as described by de Villemereuil (2012) [22]. Regarding heritability, we estimated the genetic and environmental variances according to a single-response linear mixed model:

$$y_{N\times 1}=1_{N\times 1}\mu +g_{N\times 1}+\varepsilon_{N\times 1},$$

where **y**_*N*×1_ is the vector of the mean phenotype (i.e., elemental contents) for each genotype of the length *N* (*N* = number of genotypes), **1**_*N*×1_ is the vector of ones of length *N*, *μ* is the overall mean, **g**_*N*×1_ is the vector of breeding value for each genotype, and **ε**_*N*×1_ is the vector of residual errors. The breeding values **g**_*N*×1_ and residuals **ε**_*N*×1_ were assumed to exhibit multivariate normal distributions:

$$g_{N\times 1}\sim N\left(0;\sigma_{g}^{2}A_{N\times N}\right)$$

and

$$\varepsilon_{N\times 1}\sim N\left(0;\sigma_{r}^{2}I_{N\times N}\right),$$

where **A**_*N*×*N*_ is the realized additive relationship matrix derived from genome-wide markers, $\sigma_{g}^{2}$ is the additive genetic variance, **I**_*N*×*N*_ refers to the identity matrix, and $\sigma_{r}^{2}$ is the residual variance. The realized relationship matrix **A**_*N*×*N*_ was calculated using the “A.mat” function in the R package “rrBLUP” [33]. An estimate of narrow-sense heritability (${\hat{h}}^{2}$) could be calculated as:

$${\hat{h}}^{2}=\frac{{\hat{\sigma }}_{g}^{2}}{{\hat{\sigma }}_{g}^{2}+{\hat{\sigma }}_{r}^{2}},$$

where ${\hat{\sigma }}_{g}^{2}$ is an estimate of the additive genetic variance and ${\hat{\sigma }}_{r}^{2}$ is an estimate of the residual variance. Regarding the prior distribution of variances, we assumed an inverse-Gamma distribution with the expected variance (v) of 1 and degree of belief parameter (nu) of 0.002 for both genetic and residual variances. To maintain the influence of hyperparameters consistently over all elements, *y*_*i*_ was scaled to have a mean of 0 and variance of 1. The number of MCMC cycles was set to 13,000, with a thinning interval of 10 and a burn-in period of 3,000.

To calculate the genetic correlations between locations and among elements, a multi-response Bayesian method [21] was used to estimate genetic variance and covariance. The package “MCMCglmm” [21] was also used for this calculation. The multi-response Bayesian method assumed a linear mixed model:

$$y_{2N\times 1}=x_{2N\times 2}\mu_{2\times 1}+g_{2N\times 1}+\varepsilon_{2N\times 1},$$

where **y**_2*N*×1_ is the vector of the mean phenotype (elemental contents) for each genotype of the length 2*N* in the pairs of elements (or locations). The first *N* elements of the vector correspond to the first element (or location), while the second *N* elements correspond to the second element (or location). In the equation, **μ**_2×1_ is the vector of the overall means of two elements (or locations), **x**_2*N*×2_ is the corresponding design matrix of 2*N* × 2, **g**_2*N*×1_ is the vector of the breeding value for each genotype in the pairs of elements (or locations), and **ε**_2*N*×1_ is the vector of residual errors. The breeding values **g**_2*N*×1_ and residuals **ε**_2*N*×1_ were assumed to exhibit multivariate normal distributions:

$$g_{2N\times 1}\sim N\left(0;G_{2N\times 2N}\right)$$

and

$$\varepsilon_{2N\times 1}\sim N\left(0;R_{2N\times 2N}\right),$$

where **G**_2*N*×2*N*_ and **R**_2*N*×2*N*_ are the expected covariances of the random effects and residuals, respectively. **G**_2*N*×2*N*_ and **R**_2*N*×2*N*_ have variance structures of the form:

$$G_{2N\times 2N}=V_{G\;2\times 2}\;⊗A_{N\times N}$$

and

$$R_{2N\times 2N}=V_{R\;2\times 2}\;⊗\;I_{N\times N},$$

where **V**_**G** 2×2_ is the additive genetic covariance matrix in the pairs of elements (or locations), and **V**_**R** 2×2_ is the residual covariance matrix in the pairs of elements (or locations). **A**_*N*×*N*_ and **I**_*N*×*N*_ are the same as above. We estimated genetic variances and covariances in pairs of elements (or locations) as follows:

$$V_{G\;2\times 2}\;⊗A_{N\times N}=\;\left[\begin{array}{cc} \sigma_{g1}^{2} & \sigma_{g12}\\ \sigma_{g12} & \sigma_{g2}^{2} \end{array}\right]⊗A_{N\times N}$$

We then estimated the genetic correlation (*r*) as follows:

$$r=\;\frac{{\hat{\sigma }}_{g12}}{\sqrt{{\hat{\sigma }}_{g1}^{2}\;{\hat{\sigma }}_{g2}^{2}}},$$

where ${\hat{\sigma }}_{g12}$ is an estimate of the genetic covariance between element 1 and element 2, and ${\hat{\sigma }}_{gi}^{2}$ is an estimate of the additive genetic variance associated with the element *i*.

Regarding the prior distribution of variances, we assumed an inverse-Gamma distribution with the expected variance (v) of 0.5 and degree of belief parameter (nu) of 2 for **V**_**G**_, and v = 0.5 and nu = 2 for **V**_**R**_. The number of MCMC cycles was set to 13,000, with a thinning interval of 10 and a burn-in period of 3,000.

The point estimates of *h*^*2*^ and *r* were obtained from the mean of 1,000 MCMC samples, and the 95% highest posterior density (HPD) interval of *h*^*2*^ and *r* was also based on 1,000 MCMC samples. The R function “HPDinterval” in the “lme4” package was used to obtain the 95% HPD interval.

Based on the genetic correlation matrix estimated for the six elements, we completed the cluster analysis based on the Unweighted Pair Group Method with Arithmetic Mean and checked the relationship between each element.

#### Genome-wide association study

To detect significant associations between marker genotypes and phenotypes, we completed a GWAS using the “GWAS” function in the R package “rrBLUP” [33]. For the GWAS, we used the QK-model (i.e., linear mixed model with fixed effects explaining the effect of population structure and a random effect explaining polymorphic effects) [34]. The landraces used in this study were divided into 15 sub-populations, 6 of which covered 75% of all accessions [9]. In this study, six principal components of genome-wide marker scores were included in the QK-model to avoid false positives caused by population stratification in the materials. The realized relationship matrix based on genome-wide SNP markers was calculated using the “A.mat” function. The other options were set to default values. The significance threshold was selected to maintain the false discovery rate (FDR; [35]) at less than 0.2.

We also completed a multi-response GWAS to detect marker–phenotype associations in elemental contents for use in a combination analysis of the phenotypic values obtained at the two locations. To complete the multi-response GWAS, we used SNPTEST ver. 2.5.2 (https://mathgen.stats.ox.ac.uk/genetics_software/snptest/snptest.html). In this program, the Bayes factor (BF) was used as a criterion for detecting significant associations. The BF threshold was set to 3.0 as suggested by Kass and Raftery (1995) [36]. We set the Inverse-Wishart prior according to the following options: M = (0,0), V = 0.02, c = 6, and Q = 4. In the analysis, the number of principal components explaining the population structure was set as six.

#### Genomic prediction of micronutrient concentration

To evaluate the potential of selecting micronutrient-rich genotypes, we completed the following six GS methods: genomic best linear unbiased predictor (G-BLUP), reproducing kernel Hilbert space regression (RKHS), random forest (RF), ridge regression (RR), elastic net (EN), and LASSO. For G-BLUP and RKHS, we used the “kinship.BLUP” function in the R package “rrBLUP” [33] to build prediction models. For G-BLUP, we used the function “A.mat” to obtain a realized relationship matrix. For RKHS, we employed a Gaussian kernel matrix. For RF, we used the “randomForest” function in the R package “randomForest” [37]. All parameters were set to default values. For RR, EN, and LASSO, we used the “cv.glmnet” function in the R package “glmnet” [38]. The penalty for regularization parameter (α) was 0, 0.5, and 1 for RR, EN, and LASSO, respectively. Other parameters were set to default values.

To assess the accuracy of the genomic prediction of elemental contents, we completed a 10-fold cross-validation using five replicates, and calculated the average accuracy statistic value. Regarding the accuracy statistic, we calculated the correlation coefficients between predicted and observed values (i.e., phenotypic values) for all accessions. To assess the degree of shrinkage in predicted values, we also completed regression analyses using predicted and observed values, and estimated the slope of the regression line.

## Results

### Genetic variability of elements in wheat grains

**Fig 1:**
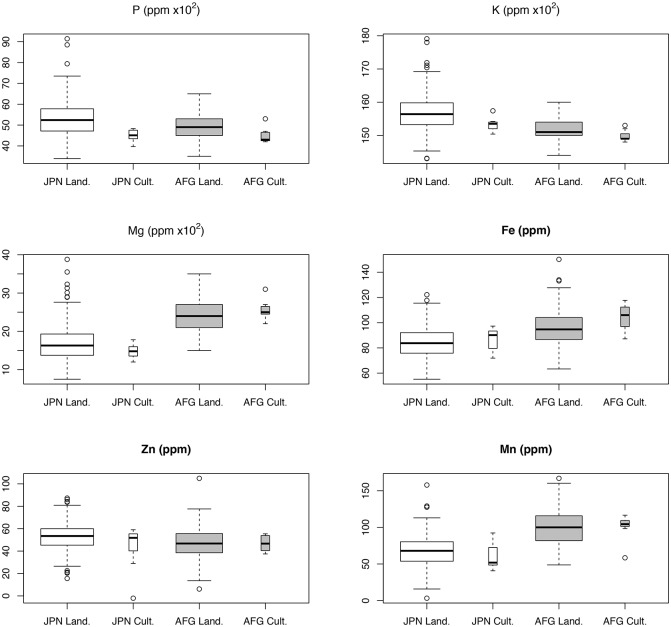
Boxplots of elemental contents in 269 Kihara Afghan wheat landraces and seven check cultivars. Results for Japan and Afghanistan are indicated in white and grey, respectively.

The range of variation for major and minor elements in 269 KAWLRs along with seven check varieties at two different locations is presented in Fig 1. Kondou et al. observed that the landraces exhibited greater variability than the check varieties regarding P, K, Mg, and Fe in Japan [29]. In the present study, we added two new elements and one new environment, and determined that the landraces were more variable and had higher average values than the check varieties for all elements except Fe at both locations. Among the major elements, K (143–179 × 10^2^ ppm) was the most available, followed by P (34–91 × 10^2^ ppm) and Mg (8–39 × 10^2^ ppm). Of the main biofortification targets, plants accumulated more Fe than Zn. However, for Fe, the average value (86.47 ppm) of the check varieties was higher than the average value (84.71 ppm) of the landraces. Outliers were detected for all elements, which increased the likelihood that germplasm with high elemental contents would be identified. Location effects were observed in all cases. Some elements (i.e., P, K, and Zn) accumulated more in plants grown in Japan, while other elements (i.e., Mg, Fe, and Mn) accumulated more in plants grown in Afghanistan.

### Heritability and correlation analysis

The narrow-sense heritabilities of elemental contents at both locations were estimated using the Bayesian linear mixed model (Table 1). Irrespective of location, the estimated heritabilities of major elements were higher than those of minor elements. Among the major elements, P was the most heritable, followed by K and Mg. Location effects strongly influenced heritability. The estimates of heritability for the samples grown in Afghanistan were lower than for plants grown in Japan, especially for the minor elements, implying the environmental variance was smaller in Japan than in Afghanistan.

**Table 1:** Marker-based heritabilities of elements.

Location	Element	*h*^*2*^ ^a^
Japan	P	0.34 (0.20, 0.50)
Japan	K	0.28 (0.13, 0.42)
Japan	Mg	0.23 (0.13, 0.36)
Japan	Fe	0.16 (0.04, 0.31)
Japan	Zn	0.24 (0.09, 0.39)
Japan	Mn	0.14 (0.001, 0.26)
Afghanistan	P	0.11 (0.002, 0.25)
Afghanistan	K	0.12 (0.002, 0.24)
Afghanistan	Mg	0.14 (0.003, 0.28)
Afghanistan	Fe	0.02 (0.000, 0.07)
Afghanistan	Zn	0.03 (0.000, 0.07)
Afghanistan	Mn	0.04 (0.000, 0.14)

^a^ Numbers inside parentheses correspond to the 95% confidence interval.

**Fig 2:**
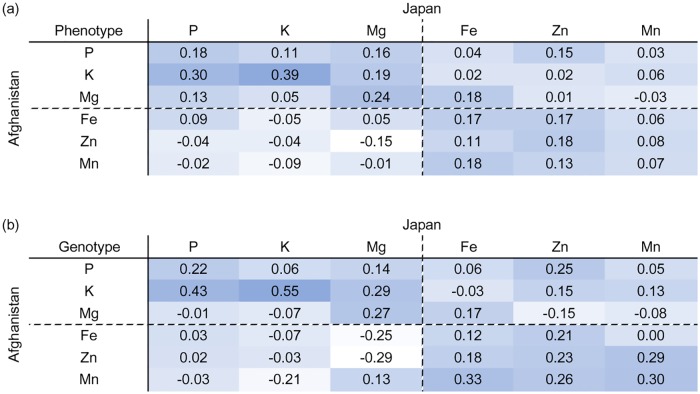
Phenotypic (a) and genetic (b) correlations between locations for all elements. The degree of correlation is indicated by blue color scale intensities.

The genetic and phenotypic correlations between locations varied among the elements (Table 2, Fig 2). Similar to the estimates of heritability, the three minor elements had low genetic correlations (0.1 < *r* ≤ 0.3). Only K had high genetic correlations (0.5 < *r*), while the other two major elements (i.e., P and Mg) exhibited low genetic correlations. Phenotypic correlations between locations were slightly smaller than the genetic correlations for most elements.

**Table 2:** Correlations between Japan and Afghanistan.

Element	Phenotypic correlation	Genotypic correlation^a^
P	0.18	0.22 (-0.15, 0.56)
K	0.39	0.55 (0.25, 0.79)
Mg	0.24	0.27 (-0.12, 0.65)
Fe	0.17	0.12 (-0.34, 0.56)
Zn	0.18	0.23 (-0.19, 0.64)
Mn	0.07	0.30 (-0.03, 0.71)

^a^ Numbers inside parentheses correspond to the 95% confidence interval.

**Fig 3:**
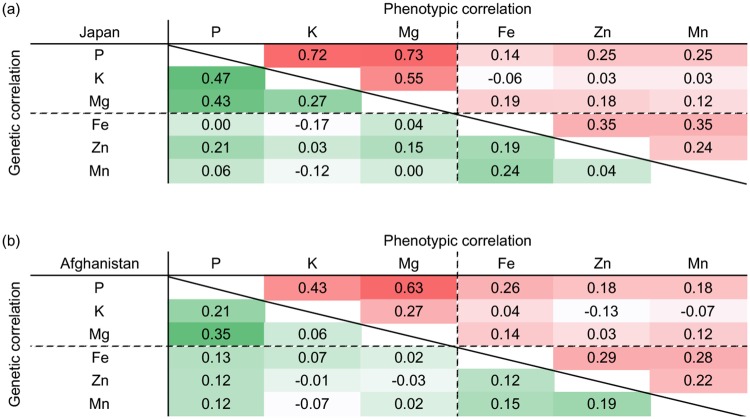
Phenotypic and genetic correlations among elements in Japan (a) and Afghanistan (b). The degree of phenotypic and genetic correlations are indicated by red and green color scale intensities, respectively.

We also estimated the genetic and phenotypic correlations among elements (Fig 3). Generally, the genetic correlations among the major elements were higher than among the minor elements. In Japan, genetic correlations among the major elements were moderate (0.3 < *r* ≤ 0.5) between P and K and P and Mg, and low (0 < *r* ≤ 0.3) between K and Mg. Genetic correlations among the minor elements were low (0 < *r* ≤ 0.3) for all possible combinations. There were no correlations between the major and minor elements except between P and Zn (*r* = 0.21). Although the genetic correlations among elements were lower in Afghanistan than in Japan, the patterns were similar. The genetic correlations among major elements were higher than those of the minor elements. There were also no genetic correlations between the major and minor elements.

**Fig 4:**
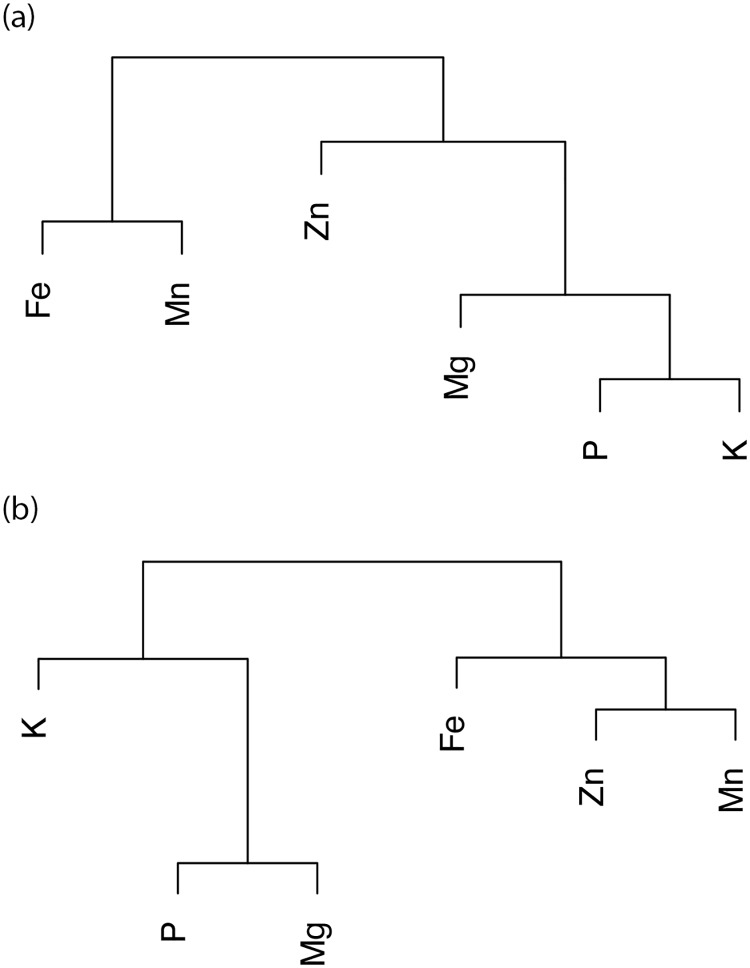
Unweighted Pair Group Method with Arithmetic Mean cluster dendrogram based on genetic correlations in Japan (a) and Afghanistan (b).

Based on the estimated genetic correlations, we completed cluster analyses to visualize the relationships among the elements (Fig 4). The dendrogram topology was different for samples grown in Japan and Afghanistan. In Japan, the P-K and Fe-Mn pairs formed terminal clusters, while in Afghanistan, P-Mg and Zn-Mn formed pairs.

### Marker-trait association

With the single-response model, the GWAS detected only one marker (1208679|F|0—64:T>C) at the FDR level of 20%. The marker was significantly associated with Zn in Afghanistan, and was located on chromosome 6D (26.98 cM). The −log10(*p*) and FDR values were 4.87 and 11.3%, respectively. In the multi-response GWAS treating phenotypes in two locations as multiple responses, no significant marker was detected at a weak BF threshold (3.0). Some markers were marginally significant. Marker 1045015|F|0 on chromosome 1A had a BF value of 2.95 for P, while marker 1017361|F|0 on chromosome 2B had a BF value of 2.77 for Mn.

### Genomic prediction

The degree of accuracy was considerably different between locations (Table 3). For all elements, the prediction models built using elemental content data from Japan were more accurate than those built using data from Afghanistan. In Japan, the genomic prediction for P was highly accurate (*r* > 0.5). The predictions were moderately accurate (0.3 < *r* ≤ 0.5) for the other two major elements (i.e., K and Mg) as well for two minor elements (i.e., Zn and Fe). The genomic predictions for Mn exhibited a low degree of accuracy (0 < *r* ≤ 0.3). Among the six methods used to build prediction models, G-BLUP, RKHS, and RF produced the most accurate predictions. Of these three methods, G-BLUP was the most accurate for P, Mg, Fe, and Mn, while RF was the most accurate for K and Zn. The RKHS predictions were almost as accurate as those of G-BLUP for all elements. The EN and LASSO methods produced the least accurate genomic predictions for all elements, suggesting that the genetic mechanism assumed by these two methods (i.e., the existence of causal genes with large additive effects) is inappropriate for the evaluated elements. The slope of the regression line for observed and predicted values was much smaller than 1.0 (Table 4), indicating the predicted values were strongly shrunk toward their average. This suggests that the predicted values contained large errors and were not credible because of their strong shrinkage nature. However, the order of the predicted values was credible because there was a correlation between the observed and predicted values.

**Table 3:** Genomic predictions of elemental contents from various models.

Location	Element	Accuracy of genomic predictions^a^^,^^b^					
		G-BLUP	RKHS	Random forest	Ridge	Elasticnet	Lasso
Japan	P	0.52 (0.02)	0.52 (0.02)	0.50 (0.01)	0.48 (0.02)	0.40 (0.01)	0.40 (0.02)
Japan	K	0.46 (0.01)	0.46 (0.01)	0.47 (0.01)	0.44 (0.05)	0.34 (0.04)	0.36 (0.01)
Japan	Mg	0.43 (0.03)	0.43 (0.03)	0.42 (0.01)	0.36 (0.11)	0.20 (0.02)	0.22 (0.06)
Japan	Fe	0.31 (0.03)	0.30 (0.03)	0.30 (0.03)	0.14 (0.03)	0.08 (0.03)	0.06 (0.07)
Japan	Zn	0.36 (0.01)	0.36 (0.02)	0.37 (0.02)	0.29 (0.06)	0.10 (0.06)	0.07 (0.05)
Japan	Mn	0.21 (0.04)	0.21 (0.04)	0.18 (0.03)	-0.08 (0.10)	-0.08 (0.08)	-0.08 (0.08)
Afghanistan	P	0.22 (0.03)	0.24 (0.04)	0.27 (0.04)	0.04 (0.05)	-0.19 (0.04)	-0.17 (0.05)
Afghanistan	K	0.23 (0.03)	0.20 (0.02)	0.24 (0.03)	0.00 (0.09)	-0.12 (0.11)	-0.09 (0.06)
Afghanistan	Mg	0.27 (0.02)	0.26 (0.04)	0.27 (0.04)	0.11 (0.07)	-0.11 (0.07)	-0.14 (0.08)
Afghanistan	Fe	-0.21 (0.06)	-0.01 (0.04)	0.02 (0.06)	-0.21 (0.06)	-0.21 (0.06)	-0.21 (0.06)
Afghanistan	Zn	-0.03 (0.03)	-0.03 (0.05)	0.11 (0.05)	-0.22 (0.03)	-0.22 (0.03)	-0.22 (0.03)
Afghanistan	Mn	0.10 (0.04)	0.10 (0.04)	0.12 (0.02)	-0.21 (0.05)	-0.19 (0.07)	-0.19 (0.07)

^a^ The most accurate predictions for each element are underlined

^b^ Numbers inside parentheses correspond to standard deviations

**Table 4:** Slopes for the genomic prediction plot (regression of predicted values on observed values).

Location	Element	Slopes of regression^a^					
		G-BLUP	RKHS	Random forest	Ridge	Elasticnet	Lasso
Japan	P	0.28 (0.01)	0.28 (0.01)	0.28 (0.00)	0.12 (0.01)	0.09 (0.01)	0.09 (0.02)
Japan	K	0.23 (0.01)	0.23 (0.00)	0.24 (0.01)	0.11 (0.02)	0.06 (0.01)	0.07 (0.01)
Japan	Mg	0.19 (0.01)	0.19 (0.01)	0.21 (0.01)	0.07 (0.03)	0.03 (0.00)	0.03 (0.02)
Japan	Fe	0.09 (0.01)	0.09 (0.01)	0.13 (0.01)	0.01 (0.00)	0.01 (0.00)	0.00 (0.00)
Japan	Zn	0.14 (0.01)	0.14 (0.01)	0.17 (0.02)	0.05 (0.02)	0.01 (0.01)	0.01 (0.01)
Japan	Mn	0.06 (0.01)	0.06 (0.01)	0.07 (0.01)	0.00 (0.00)	0.00 (0.00)	0.00 (0.00)
Afghanistan	P	0.05 (0.01)	0.06 (0.01)	0.12 (0.02)	0.00 (0.00)	-0.01 (0.00)	0.00 (0.00)
Afghanistan	K	0.07 (0.01)	0.06 (0.01)	0.11 (0.02)	0.00 (0.01)	0.00 (0.00)	-0.01 (0.00)
Afghanistan	Mg	0.08 (0.01)	0.08 (0.01)	0.12 (0.02)	0.01 (0.01)	0.00 (0.00)	0.00 (0.01)
Afghanistan	Fe	-0.01 (0.00)	0.00 (0.01)	0.01 (0.02)	-0.01 (0.00)	-0.01 (0.00)	-0.01 (0.00)
Afghanistan	Zn	0.00 (0.00)	0.00 (0.01)	0.04 (0.02)	-0.01 (0.00)	-0.01 (0.00)	-0.01 (0.00)
Afghanistan	Mn	0.02 (0.01)	0.02 (0.01)	0.05 (0.01)	-0.01 (0.00)	0.00 (0.00)	-0.01 (0.00)

^a^ Numbers inside parentheses correspond to standard deviations

In Afghanistan, none of the genomic predictions were highly (*r* > 0.5) or even moderately (0.3 < *r* ≤ 0.5) accurate. The accuracy of the predictions for the three major elements (i.e., P, K, and Mg) as well as for two minor elements (i.e., Mn and Zn) was low (0 < *r* ≤ 0.3). There was no correlation between the observed and predicted elemental contents for Fe. Similar to the results in Japan, of the six methods used, G-BLUP, RKHS, and RF produced the most accurate predictions in Afghanistan. Additionally, RF was the most accurate for all elements. The G-BLUP method exhibited the same degree of accuracy as RF for Mg, and this method was the most accurate for Mg in Japan. Similar to what was observed in Japan, the accuracy of the RKHS predictions in Afghanistan was very similar to that of the G-BLUP predictions. The EN and LASSO predictions were the least accurate for all elements, which was consistent with the results in Japan. For all elements, EN and LASSO indicated the observed and predicted values for elemental contents were negatively correlated. The slope of the regression line for observed and predicted values was less than 0.1 for all elements (Table 4), indicating the predicted values were more strongly shrunk toward their average in Afghanistan than in Japan.

## Discussion

Information regarding the genetic nature of a particular trait is necessary for efficient genetic improvement of crops. Elements are the basic unit of biological organisms, and their enrichment (i.e., biofortification) is an important crop improvement objective. Most previous studies on the elemental contents of wheat grains focused on Fe and Zn [4, 8, 25, 39]. These two elements are often lacking in the human diet, which makes them important biofortification targets [40–41]. There is often a correlation between elements, which might imply there are elemental interactions during plant metabolic activities [1]. It may be worthwhile to investigate the movement of other major and minor elements to increase the concentration of micronutrients such as Fe and Zn. In addition to Fe and Zn, deficiencies in other micronutrients, such as Ca, Mg, and Cu, are common in many developed and developing countries [42]. In the present study, we observed low to high genetic correlations among elements, suggesting the utility of integrated analyses of multiple elements.

Accessions producing grains with high micronutrient concentrations are often identified in landraces, wild wheat, and their relatives [43–44]. The results of our elemental analyses of Afghan landraces were consistent with the findings of these earlier studies. For example, the concentrations of Fe and Zn in this study were 55.14–122.2 ppm and 15.56–87.29 ppm, respectively, which were much higher than the concentrations (Fe: 28.8–56.5 ppm; Zn: 25.2–53.3 ppm) reported for 132 cultivars screened by the International Maize and Wheat Improvement Center (CIMMYT) [45]. Our concentrations were also higher than those of 150 lines of bread wheat representing diverse origins (Fe: 28.8–50.8 ppm; Zn: 13.5–34.5 ppm; [46]). The Afghan landraces seemed to have higher concentrations of the major elements than the cultivars screened by CIMMYT [45]. These results suggest that some Afghan landraces may represent good genetic resources for improving grain micronutrient concentrations. However, when comparing results from different studies, it is important to acknowledge that environmental factors, such as soil nutrient composition, can considerably influence the elemental contents of plants. Because of the practical difficulty of acquiring soil samples from Afghanistan, soil analysis for the comparison of elemental uptake was not performed in this study. In the future, it may be possible to establish a soil-analysis laboratory in vivo and to carry out the analysis in conjunction with an associated technical cooperative project.

In the present study, we performed field experiments in Japan and Afghanistan using the same set of Afghan wheat landraces. We observed similar trends between the two locations, suggesting the potential of the Afghan landraces to be grown outside of Afghanistan. The genetic nature of the elemental contents was also confirmed at both locations. However, there were clear location effects, with plants accumulating more P, K, and Zn in Japan than in Afghanistan, and *vice versa* for the other elements. Although the experiments were conducted with uniform nutrient management practices, the soil type and nutrient content are important factors to consider. The soils of Afghanistan are alkaline, with 50% of the soils having a pH between 8 and 8.5. They are also generally rich in alkaline earth carbonates [47]. Calcareous soils are usually lacking in P, Zn, and Fe (FAO Soils Portal, 2016; http://www.fao.org/soils-portal/en/). These soil conditions may have been responsible for the observed differences in the elemental contents of plants grown in Japan and Afghanistan.

The genetic nature of elemental contents was assessed by estimating marker-based heritabilities and genetic correlations among elements and between locations. For this approach, genetic variance and covariance were estimated according to the realized relationship matrix derived from marker genotypes [21], and were used to calculate heritability and genetic correlation [22, 48]. The estimated heritabilities and genetic correlations for the two locations were higher for the major elements than for the minor elements, suggesting it may be possible to detect germplasm with high major element contents irrespective of where the plants are grown. Identifying ideal germplasm with high minor element concentrations may be more difficult because of the observed low heritabilities and genetic correlations for the two locations. The fact that estimated heritabilities were lower in Afghanistan than in Japan suggests the environmental stresses in Afghanistan produced larger environmental variances than the stresses in Japan. The correlation and the clustering analyses based on the correlation matrix revealed inter-relationships among the major elements and among the minor elements. These results imply that the same genetic and/or physiological mechanisms are shared among the major elements and among the minor elements for the uptake and/or accumulation of elements. Additionally, the Fe and Zn concentrations were positively correlated, which is consistent with the findings of other studies involving wheat [39, 46].

In the present study, the only significant marker detected in the single-response GWAS was located on chromosome 6D and was associated with Zn. Although we used a relatively high FDR threshold (0.2), no significant association was detected except for the marker associated with Zn. This result indicates the potential difficulty in identifying major quantitative trait loci for elemental contents in wheat grains through a GWAS. In this study, the detection power of the multi-response GWAS was similar to that of the single-response GWAS. That is, significant markers were not detected even with a weak BF threshold of 3.0 [36], suggesting that identifying markers significantly associated with elemental contents in wheat grains will be challenging. The low power of the GWAS was probably due to small sample size, low LD between markers and causal variants, low frequencies of causal variants [49], adjustment for the confounding effect of population stratification [50], and the low heritability of elemental contents. The increment of sample size and marker number and careful selection of materials [50] are expected to improve the power of GWAS for elemental contents.

Only one study that assessed the accuracy of genomic predictions of elemental contents in crop plants has been published [27]. Because the elemental contents of wheat grains are influenced by environmental conditions, as suggested in the present study, the accuracy of genomic predictions of elemental contents requires further empirical research to confirm the potential of GS for improving nutrient contents in wheat grains. Based on the results obtained in the present study, we determined that genomic predictions were moderately-to-highly accurate for most of the elements measured in Japan, even though no significant association was detected in the single-trait GWAS except for the one detected for Zn. These results suggest that the elemental contents are mainly controlled by a number of genes with small-to-moderate effects. This is supported by the fact that genomic predictions from LASSO and EN, which assume some independent variables (i.e., markers) have large effects on a dependent variable (i.e., phenotype of a trait), were less accurate than those of the other methods. The genomic predictions were more accurate for the major elements than for the minor elements. They were also more accurate in Japan than in Afghanistan. The accuracy of the genomic predictions was related to the degree of heritability of each element at each location, with greater accuracy being associated with higher heritability. When the correlation coefficient was divided by the square root of the heritability value, the resulting accuracies were relatively high for the major elements (0.7–0.9), but low for the minor elements (0.4–0.7, except for Fe in Afghanistan) at both locations (Table 5). This result may indicate that the major elements can be improved more efficiently with GS than the minor elements. The accuracy of the genomic predictions for Fe in Afghanistan was low (≤ 0.1) even after the adjustment.

**Table 5:** Adjusted accuracies of genomic predictions.

Location	Element	Model^a^	Accuracy^a^
Japan	P	G-BLUP, RKHS	0.90
Japan	K	G-BLUP, RKHS, Random forest	0.88
Japan	Mg	G-BLUP	0.90
Japan	Fe	G-BLUP	0.77
Japan	Zn	Random forest	0.75
Japan	Mn	G-BLUP	0.57
Afghanistan	P	Random forest	0.82
Afghanistan	K	Random forest	0.70
Afghanistan	Mg	G-BLUP	0.72
Afghanistan	Fe	Random forest	0.11
Afghanistan	Zn	Random forest	0.65
Afghanistan	Mn	Random forest	0.60

^a^ Results for the most accurate model (of the six analyzed: G-BLUP, RKHS, Random forest, Ridge, Elasticnet, and Lasso) are presented

We used six methods to determine the accuracy of the genomic predictions. The RF was one of the most accurate methods, especially for the minor elements or when the heritability of elemental contents was low (i.e., in Afghanistan). These findings are consistent with those of other studies [51–52]. In the simulations described by Onogi et al. (2016) [51], the RF method was superior when there was low heritability, a small training set, or epistasis. The accuracy of the G-BLUP and RKHS genomic predictions were similar for most elements. Because G-BLUP and RKHS are linear and non-linear kernel regression methods, respectively, our findings imply that non-linearity between marker genotypes and phenotypes does not dominate the inheritance of elemental contents (i.e., the inheritance of elemental contents is mainly controlled by additive genetic effects). Our data may also indicate that the superiority of RF is mainly the result of low heritability rather than the existence of epistatic effects. However, it is possible that the RKHS and RF methods may not have fully captured the non-linearity between marker genotypes and phenotypes. Because studies related to the genomic prediction of elemental contents are not available in the literature, we cannot compare our data with those of other studies. Future studies will need to assess the potential of GS for breeding biofortified wheat cultivars.

Even for the elements in which the accuracy of genomic predictions was low, there are still the following advantages of GS over phenotypic selection: (1) The time and cost required for phenotypic selection are greater than those for GS. Replications (i.e., plants and plots) are necessary for phenotyping, but not for genotyping. Additionally, plants must be cultivated in a field for phenotyping, but not for genotyping, which eliminates the associated costs. (2) Selection cycles can be accelerated during GS breeding. Phenotyping can only be completed once per growing season because elemental contents can be affected by cultivation conditions. Therefore, phenotypic selection should take place under consistent environmental conditions. In contrast, time and location do not influence genotyping. Thus, rapid generation advancement technologies can be used for GS breeding. (3) Segregation of target traits in the next generation and the improvement of target traits in future generations can be simulated during GS breeding [53–54]. The results of these simulations can then be used to optimize breeding procedures before completing selection and crossing experiments. They can also be used to estimate the expected genetic gain. Moreover, GS enables the development of new breeding materials for Afghanistan in other countries by combining the results of genomic prediction models and field trial data from Afghanistan. This “remote breeding” approach can use Afghan wheat landraces to improve the quality of wheat produced in Afghanistan. Multi-year and multi-location field trial data will enhance the accuracy of genomic predictions and genetic gain from GS. Further research is necessary to improve the accuracy of predictions regarding elemental contents, and to develop a remote breeding system based on GS.

## Conclusions

The potential of Afghan wheat landraces for increasing grain elemental contents was analyzed in this study. An EDXRF-based high-throughput method was used to analyze 269 landraces for six elements. The measured elements showed varied genetic nature, with major elements exhibiting higher heritability than minor elements. The lack of a significant correlation between major and minor elements suggests that genetic systems are independent between major and minor elements. A GWAS revealed only one significant marker-trait association. The low power of the GWAS could probably be improved by a larger sample size and a larger number of markers. Although the GWAS detected no significant association for most elements, genomic predictions for elemental contents were moderately to highly accurate. Based on our results, the possibility of establishing a “remote breeding” approach involving genomic selection is under discussion.

## Supporting Information

S1 Table Passport details of Afghan wheat landraces. ^a^ Variety names are shown for seven check varieties ^b^ National Bio-Resource Project, Japan.
(PDF)

S2 Table Marker genotype and map position data. (XLSX)

S3 Table Elemental content data. (XLSX)

## References

[1] Baxter I. Ionomics: The functional genomics of elements. Briefings in Functional Genomics. 2010; 9 (2): 149–156. 10.1093/bfgp/elp055 20081216

[2] Salt DE, Baxter I, Lahner B. Ionomics and the study of the plant ionome. Annual Review of Plant Biology. 2008; 59: 709–33. 10.1146/annurev.arplant.59.032607.092942 18251712

[3] World Health Organization. The World Health Report. 2013.

[4] Welch RM, Graham RD. Breeding for micronutrients in staple food crops from a human nutrition perspective. J Exp Bot. 2004; 55 (396): 353–364. 10.1093/jxb/erh064 14739261

[5] Bouis HE. The potential of genetically modified food crops to improve human nutrition in developing countries. The Journal of Development Studies. 2007; 43 (1): 79–96.

[6] Cakmak I. Enrichment of cereal grains with zinc: agronomic or genetic biofortification? Plant and Soil. 2008; 302 (1): 1–17.

[7] Peleg Z, Cakmak I, Ozturk L, Yazici A, Jun Y, Budak H, et al Quantitative trait loci conferring grain mineral nutrient concentrations in durum wheat × wild emmer wheat RIL population. Theor Appl Genet. 2009; 119 (2): 353–369. 10.1007/s00122-009-1044-z 19407982

[8] Velu G, Ortiz-Monasterio I, Cakmak I, Hao Y, Singh RP. Biofortification strategies to increase grain zinc and iron concentrations in wheat. Journal of Cereal Science. 2014; 59 (3): 365–372.

[9] Manickavelu A, Jighly A, Ban T. Molecular evaluation of orphan Afghan common wheat (*Triticum aestivum* L.) landraces collected by Dr. Kihara using single nucleotide polymorphic markers. BMC Plant Biology. 2014; 14: 320 10.1186/s12870-014-0320-5 25432399 PMC4255927

[10] Manickavelu A, Niwa S, Ayumi K, Komatsu K, Naruoka Y, Ban T. Molecular evaluation of Afghanistan wheat landraces. Plant Genetic Resources: Characterization and Utilization. 2014; 12 (S1): S31–S35.

[11] Sohail Q, Manickavelu A, Ban T. Genetic diversity analysis of Afghan wheat landraces (*Triticum aestivum*) using DArT markers. Genet Resour Crop Evol. 2015; 62 (8): 1147–1157.

[12] Glaszmann JC, Kilian B, Upadhyaya HD, Varshney RK. Accessing genetic diversity for crop improvement. Current Opinion in Plant Biology. 2010; 13 (2): 167–173. 10.1016/j.pbi.2010.01.004 20167531

[13] Myles S, Peiffer J, Brown PJ, Ersoz ES, Zhang Z, Costich DE, et al Association mapping: critical considerations shift from genotyping to experimental design. The Plant Cell. 2009; 21 (8): 2194–2202. 10.1105/tpc.109.068437 10.1105/tpc.109.068437 19654263 PMC2751942

[14] Meuwissen THE, Hayes BJ, Goddard ME. Prediction of total genetic value using genome-wide dense marker maps. Genetics. 2001; 157 (4): 1819–1829. 11290733 10.1093/genetics/157.4.1819 PMC1461589

[15] Desta ZA, Ortiz R. Genomic selection: genome-wide prediction in plant improvement. Trends in Plant Science. 2014; 19 (9): 592–601. 10.1016/j.tplants.2014.05.006 24970707

[16] Bernardo R, Yu J. Prospects for genome wide selection for quantitative traits in maize. Crop Sci. 2006; 47 (3): 1082–1090.

[17] Heffner EL, Sorrells ME, Jannink JL. Genomic selection for crop improvement. Crop Sci. 2008; 49 (1): 1–12.

[18] Jannink JL, Lorenz AJ, Iwata H. Genomic selection in plant breeding: from theory to practice. Briefings in Functional Genomics. 2010; 9 (2): 166–177. 10.1093/bfgp/elq001 20156985

[19] Lorenz AJ, Smith KP, Jannink JL. Potential and optimization of genomic selection for fusarium head blight resistance in six-row barley. Crop Sci. 2011; 52 (4): 1609–1621.

[20] Goddard ME, Hayes BJ. Genomic selection. Journal of Animal Breeding and Genetics. 2007; 124 (6): 323–330. 10.1111/j.1439-0388.2007.00702.x 18076469

[21] Hadfield J. MCMC methods for multi-response generalized linear mixed models: The MCMCglmm R package. Journal of Statistical Software. 2010; 33: 1–22. 20808728 PMC2929880

[22] de Villemereuil P, Wells JA, Edwards RD, Blomberg SP. Bayesian models for comparative analysis integrating phylogenetic uncertainty. BMC Evolutionary Biology. 2012; 12: 102 10.1186/1471-2148-12-102 22741602 PMC3582467

[23] Paltridge NG, Milham PJ, Ortiz-Monasterio JI, Velu G, Yasmin Z, Palmer LJ, et al Energy-dispersive X-ray fluorescence spectrometry as a tool for zinc, iron and selenium analysis in whole grain wheat. Plant Soil. 2012; 361 (1): 261–269.

[24] Velu G, Singh RP, Huerta-Espino J, Peña-Bautista RJ, Ortiz-Monasterio I. Breeding for enhanced zinc and iron concentration in CIMMYT spring wheat germplasm. Czech Journal of Genetics and Plant Breeding. 2011; 47: S174–S177.

[25] Velu G, Singh RP, Huerta-Espino J, Peña RJ, Arun B, Mahendru-Singh A, et al Performance of biofortified spring wheat genotypes in target environments for grain zinc and iron concentrations. Field Crops Research. 2012; 137: 261–267

[26] Shakoor N, Ziegler G, Dilkes BP, Brenton Z, Boyles R, Connolly EL, Kresovich S, Baxter IR (2016) Integration of experiments across diverse environments identifies the genetic determinants of variation in Sorghum bicolor seed element composition. Plant Phy 170:1989–1998 10.1104/pp.15.01971 PMC4825124 26896393

[27] Velu G, Crossa J, Singh RP, Hao Y, Dreisigacker S, Perez-Rodriguez P, et al Genomic prediction for grain zinc and iron concentrations in spring wheat. Theor Appl Genet. 2016; 129 (8): 1595–1605. 10.1007/s00122-016-2726-y 27170319

[28] Onogi A, Ideta O, Inoshita Y, Ebana K, Yoshioka T, Yamasaki M, et al Exploring the areas of applicability of whole-genome prediction methods for Asian rice (*Oryza sativa* L.). Theor Appl Genet. 2015; 128 (1): 41–53. 10.1007/s00122-014-2411-y 25341369

[29] Kondou Y, Manickavelu A, Komatsu K, Arifi M, Kawashima M, Ishii T, et al Analysis of grain elements and identification of best genotypes for Fe and P in Afghan wheat landraces. Breeding Science. 10.1270/jsbbs.16041 PMC5282750 28163583

[30] Akbari M, Wenzl P, Caig V, Carling J, Xia L, Yang S, et al Diversity arrays technology (DArT) for high-throughput profiling of the hexaploid wheat genome. Theor Appl Genet. 2006; 113 (8): 1409–1420. 10.1007/s00122-006-0365-4 17033786

[31] Wenzl P, Carling J, Kudrna D, Jaccoud D, Huttner E, Kleinhofs A, et al Diversity arrays technology (DArT) for whole-genome profiling of barley. PNAS. 2004; 101 (26): 9915–9920. 10.1073/pnas.0401076101 15192146 PMC470773

[32] Browning BL, Browning SR. A unified approach to genotype imputation and haplotype phase inference for large data sets of trios and unrelated individuals. The American Journal of Human Genetics. 2009; 84 (2): 210–223. 10.1016/j.ajhg.2009.01.005 19200528 PMC2668004

[33] Endelman JB. Ridge regression and other kernels for genomic selection with R package rrBLUP. Plant Genome. 2011; 4 (3): 250–255.

[34] Yu J, Pressoir G, Briggs WH, Vroh I, Yamasaki M, Doebley JF, et al A unified mixed-model method for association mapping that accounts for multiple levels of relatedness. Nature Genetics. 2005; 38: 203–208. 10.1038/ng1702 16380716

[35] Benjamini Y, Hochberg Y. Controlling the false discovery rate: a practical and powerful approach to multiple testing. Journal of Royal Statistical Society. 1995; 57 (1): 289–300.

[36] Kass RE, Raftery AE. Bayes factors. Journal of the American Statistical Association. 1995; 90: 773–795.

[37] Breiman L. Random forests. Machine Learning. 2001; 45 (1): 5–32.

[38] Friedman J, Hastie T, Tibshirani R. Regularization paths for generalized linear models via coordinate descent. Journal of Statistical Software. 2010; 33 (1): 1–22. 20808728 PMC2929880

[39] Morgounov A, Goez-Becerra HF, Abugalieva A, Dzhunusova M, Yessimbekova M, Muminjanov H, et al Iron and zinc grain density in common wheat grown in Central Asia. Euphytica. 2007; 155 (1): 193–203.

[40] Welch RM, Graham RD. A new paradigm for world agriculture: meeting human needs: productive, sustainable, nutritious. Field Crops Research. 1999; 60 (1–2): 1–10.

[41] Bouis HE, Welch RM. Biofortification—a sustainable agricultural strategy for reducing micronutrient malnutrition in the global South. Crop Sci. 2010; 50: S20–S32.

[42] White PJ, Broadley MR. Biofortification of crops with seven mineral elements often lacking in human diets—iron, zinc, copper, calcium, magnesium, selenium and iodine. New Phytologist. 2009; 182 (1): 49–84. 10.1111/j.1469-8137.2008.02738.x 19192191

[43] White PJ, Broadley MR. Biofortifying crops with essential mineral elements. Trends in Plant Science. 2005; 10 (12): 586–93. 10.1016/j.tplants.2005.10.001 16271501

[44] Ortiz-Monasterio JI, Palacios-Rojas N, Meng E, Pixley K, Trethowan R, Peña RJ. Enhancing the mineral and vitamin content of wheat and maize through plant breeding. Journal of Cereal Science. 2007; 46 (3): 293–307.

[45] Graham R, Senadhira D, Beebe S, Iglesias C, Monasterio I. Breeding for micronutrient density in edible portions of staple food crops conventional approaches. Field Crops Research. 1999; 60 (1–2): 57–80.

[46] Zhao FJ, Su YH, Dunham SJ, Rakszegi M, Bedo Z, McGrath SP, et al Variation in mineral micronutrient concentrations in grain of wheat lines of diverse origin. Journal of Cereal Science. 2009; 49 (2): 290–295.

[47] FAO Soil bulletin. Report based on FAO/UNDP regional seminar on reclamation and management of calcareous soils. 1973.

[48] Beaulieu J, Doerksen TK, MacKay J, Rainville A, Bousquet J. Genomic selection accuracies within and between environments and small breeding groups in white spruce. BMC Genomics. 2014; 15: 1048 10.1186/1471-2164-15-1048 25442968 PMC4265403

[49] Yang J, Benyamin B, McEvoy BP, Gordon S, Henders AK, Nyholt DR, et al Common SNPs explain a large proportion of the heritability for human height. Nature Genetics. 2010; 42: 565–569. 10.1038/ng.608 20562875 PMC3232052

[50] Brachi B, Morris GP, Borevitz JO. Genome-wide association studies in plants: the missing heritability is in the field. Genome Biology. 2011; 12: 232 10.1186/gb-2011-12-10-232 22035733 PMC3333769

[51] Onogi A, Watanabe M, Mochizuki T, Hayashi T, Nakagawa H, Hasegawa T, et al Toward integration of genomic selection with crop modelling: the development of an integrated approach to predicting rice heading dates. Theor Appl Genet. 2016; 129 (4): 805–817. 10.1007/s00122-016-2667-5 26791836

[52] Spindel J, Begum H, Akdemir D, Virk P, Collard B, Redoña E, et al Genomic selection and association mapping in rice (*Oryza sativa*): effect of trait genetic architecture, training population composition, marker number and statistical model on accuracy of rice genomic selection in elite, tropical rice breeding lines. PLoS Genet. 2015; 11 (2): e1004982 10.1371/journal.pgen.1004982 25689273 PMC4334555

[53] Iwata H, Hayashi T, Terakami S, Takada N, Sawamura Y, Yamamoto T. Potential assessment of genome-wide association study and genomic selection in Japanese pear *Pyrus pyrifolia*. Breeding Science. 2013; 63 (1): 125–140. 10.1270/jsbbs.63.125 23641189 PMC3621438

[54] Yamamoto E, Matsunaga H, Onogi A, Kajiya-Kanegae H, Minamikawa M, Suzuki A, et al A simulation-based breeding design that uses whole-genome prediction in tomato. Scientific Reports. 2016; 6: 19454 10.1038/srep19454 26787426 PMC4726135

