# Supplementary material for: Genetic Nature of Elemental Contents in Wheat Grains and Its Genomic Prediction: Toward the Effective Use of Wheat Landraces from Afghanistan
Source: PLoS One. 2017 Jan 10;12(1):e0169416. doi: 10.1371/journal.pone.0169416 (PMC5224831; doi:10.1371/journal.pone.0169416)
Supplement: S1 Table — a Variety names are shown for seven check varieties b National Bio-Resource Project, Japan. (PDF) [file pone.0169416.s001.pdf]

Entry No.	Gene bank accession No.[a]	Collection site	Province	Agro-climatic zone	Longitude	Latitude	Altitude by NBRP (MSL)[b]	Storage year	Field trial in Japan	Field trial in Afghanistan
502	KU11202A	Bazar of Kabul	Kabul	F	69.171703	34.528455	-	1979	○	○
504	KU11202Bb	Bazar of Kabul	Kabul	F	69.171703	34.528455	-	1979	○	○
507	KU11205	8 km N of Kunduz to Eman Sahab	Kunduz	B	68.80188	36.79609	-	1979	○	○
508	KU11206	Chinzaii E of Khanabad Takhar	Takhar	B	69.112244	36.702559	640	1979	○	-
509	KU11207	Chinzaii E of Khanabad Takhar	Takhar	B	69.112244	36.702559	640	1979	○	○
511	KU11208B	Chinzaii E of Khanabad Takhar	Takhar	B	69.112244	36.702559	640	1979	○	○
512	KU11209	Chinzaii E of Khanabad Takhar	Takhar	B	69.112244	36.702559	640	1979	○	○
515	KU11212	13.2 km S of Farha Takhar	Takhar	B	62.1123	32.3754	1300	1979	○	○
516	-	-	-	B	62.1123	32.3754	-	-	○	○
517	KU11214	37 km E of Taluquan near Kalafgan Takhar	Takhar	B	69.9026	36.7631	1370	1979	○	○
518	KU11215	37 km E of Taluquan near Kalafgan Takhar	Takhar	B	69.9026	36.7631	1370	1979	○	○
519	KU11216A	37 km E of Taluquan near Kalafgan Takhar	Takhar	B	69.9026	36.7631	1370	1979	○	○
521	KU11217	41 km E of Taluquan near Karafgan Takhar	Takhar	B	69.529724	36.79609	1530	1979	○	○
523	KU11219	47 km E of Taluquan near Karafgan Takhar	Takhar	B	69.529724	36.79609	1580	1979	○	○
524	KU11221A	6 km W of Kisim Badakhshan	Badakhshan	B	70.811995	36.734772	1060	1979	○	○
525	KU11221B	6 km W of Kisim Badakhshan	Badakhshan	B	70.811995	36.734772	1060	1979	○	○
526	KU11222	6 km W of Kisim Badakhshan	Badakhshan	B	70.811995	36.734772	1060	1979	○	○
528	KU11224A	6 km W of Kisim Badakhshan	Badakhshan	B	70.811995	36.734772	1060	1979	○	○
530	KU11225	8 km S of Faizabad Badakhshan	Badakhshan	B	70.5132	37.147	1230	1979	○	○
534	KU11229	16 km SE of Faizabad Badakhshan	Badakhshan	B	70.5132	37.147	1300	1979	○	○
536	KU11230B	16 km SE of Faizabad Badakhshan	Badakhshan	B	70.5132	37.147	1300	1979	○	○
537	KU11231	Sarai Jamarad 13 km NW of Barak Badakhshan	Badakhshan	B	66.461792	35.101934	1390	1979	○	-
539	KU11233	8 km NW of Barak Badakhshan	Badakhshan	B	69.6	35.35	1450	1979	○	-
542	KU11236A	8 km NW of Barak Badakhshan	Badakhshan	B	69.6	35.35	1450	1979	○	○
543	KU11236B	8 km NW of Barak Badakhshan	Badakhshan	B	69.6	35.35	1450	1979	○	○
544	KU11237A	Puli-zeribon Shewa Badakhshan	Badakhshan	B	70.488281	33.266824	2540	1979	○	○
545	KU11237B	Puli-zeribon Shewa Badakhshan	Badakhshan	B	70.488281	33.266824	2540	1979	○	○
548	KU11239Ab	Puli-zeribon Shewa Badakhshan	Badakhshan	B	70.488281	33.266824	2540	1979	○	○
549	KU11240A	Bamdara 13 km N of Robobi to Shewa Badakhshan	Badakhshan	B	68.9377778	35.9277778	1680	1979	○	○
551	KU11241A	Bamdara 13 km N of Robobi to Shewa Badakhshan	Badakhshan	B	68.9377778	35.9277778	1680	1979	○	○
552	KU11241B	Bamdara 13 km N of Robobi to Shewa Badakhshan	Badakhshan	B	68.9377778	35.9277778	1680	1979	○	○
556	KU11243B	Bamdara 13 km N of Robobi to Shewa Badakhshan	Badakhshan	B	68.9377778	35.9277778	1680	1979	○	○
558	KU11245	Yardar 4 km SE of Barak Badakhshan	Badakhshan	B	70.883789	37.753344	1430	1979	○	-
559	KU11246	Yardar ca. 5 km SE of Barak Badakhshan	Badakhshan	B	70.883789	37.753344	1460	1979	○	○
560	KU11247	Yardar ca. 5 km SE of Barak Badakhshan	Badakhshan	B	70.883789	37.753344	1460	1979	○	○
562	KU11249A	Yardar ca. 5 km SE of Barak Badakhshan	Badakhshan	B	70.883789	37.753344	1460	1979	○	○
563	KU11249B	Yardar ca. 5 km SE of Barak Badakhshan	Badakhshan	B	70.883789	37.753344	1460	1979	○	○
564	KU11250A	6 km S of Barak Badakhshan	Badakhshan	B	69.6	35.35	1460	1979	○	○
566	KU11251	6 km S of Barak Badakhshan	Badakhshan	B	69.6	35.35	1460	1979	○	○
567	KU11252A	6 km S of Barak Badakhshan	Badakhshan	B	69.6	35.35	1460	1979	○	○
568	KU11252B	6 km S of Barak Badakhshan	Badakhshan	B	69.6	35.35	1460	1979	○	-
572	KU11255A	13 km S of Barak Badakhshan	Badakhshan	B	69.6	35.35	1460	1979	○	○

578	KU11259	Chinge Peyong 2.5 km N of Jurum Badakhshan	Badakhshan	B	70.9613889	34.1077778	1440	1979	○	-
579	KU11260	Chinge Peyong 2.5 km N of Jurum Badakhshan	Badakhshan	B	70.9613889	34.1077778	1440	1979	○	○
580	KU11261	Chinge Peyong 2.5 km N of Jurum Badakhshan	Badakhshan	B	70.9613889	34.1077778	1440	1979	○	-
582	KU11263	Khanadab 2.5 km S of Jurum Badakhshan	Badakhshan	B	69.112244	36.702559	-	1979	○	-
584	KU11265A	Khanadab 2.5 km S of Jurum Badakhshan	Badakhshan	B	69.112244	36.702559	-	1979	○	-
585	KU11265B	Khanadab 2.5 km S of Jurum Badakhshan	Badakhshan	B	69.112244	36.702559	-	1979	○	○
586	KU11266	Khanadab 2.5 km S of Jurum Badakhshan	Badakhshan	B	69.112244	36.702559	-	1979	○	-
588	KU11268	5 km S of Jurum Badakhshan	Badakhshan	B	70.82	36.849998	1490	1979	○	○
589	KU11269	5 km S of Jurum Badakhshan	Badakhshan	B	70.82	36.849998	1490	1979	○	○
590	KU11270	5 km S of Jurum Badakhshan	Badakhshan	B	70.82	36.849998	1490	1979	○	○
595	KU11274	North of Jurum Badakhshan	Badakhshan	B	70.82	36.849998	1570	1979	○	○
596	KU11275	North of Jurum Badakhshan	Badakhshan	B	70.82	36.849998	1570	1979	○	-
599	KU11278A	1.5 km E of Barak Badakhshan	Badakhshan	B	69.6	35.35	1500	1979	○	-
600	KU11278B	1.5 km E of Barak Badakhshan	Badakhshan	B	69.6	35.35	1500	1979	○	○
605	KU11282A	1.5 km E of Barak Badakhshan	Badakhshan	B	69.6	35.35	1500	1979	○	○
611	KU11286	2.5 km E of Barak Badakhshan	Badakhshan	B	69.6	35.35	1540	1979	○	○
612	KU11287	2.5 km E of Barak Badakhshan	Badakhshan	B	69.6	35.35	1540	1979	○	○
613	KU11288	2.5 km E of Barak Badakhshan	Badakhshan	B	69.6	35.35	1540	1979	○	○
616	KU11291	Pijoj 13 km E of Barak Badakhshan	Badakhshan	B	66.461792	35.101934	1750	1979	○	○
618	KU11293	20 km E of Barak Badakhshan	Badakhshan	B	69.6	35.35	1800	1979	○	-
620	KU11295A	Rezwon 27 km E of Barak Badakhshan	Badakhshan	B	66.461792	35.101934	1940	1979	○	-
628	KU11299	Rezwon 27 km E of Barak Badakhshan	Badakhshan	B	66.461792	35.101934	1940	1979	○	-
649	KU11316	Yomal 34 km SE of Barak Badakhshan	Badakhshan	B	66.461792	35.101934	1810	1979	○	-
652	KU11318	Tirgaran 46 km SE of Barak Badakhshan	Badakhshan	B	70.9833333	36.1791666	2040	1979	○	○
658	KU11323	Tirgaran ca. 49 km SE of Barak to Zebak Badakhshan	Badakhshan	B	70.9833333	36.1791666	2120	1979	○	○
664	KU11328	Shakelan 33 km SE of Barak to Zebak Badakhshan	Badakhshan	B	71.38916	36.93233	1650	1979	○	○
665	KU11329	Shakelan 33 km SE of Barak to Zebak Badakhshan	Badakhshan	B	71.38916	36.93233	1650	1979	○	○
666	KU11330	Awan 25 km SE of Barak to Zebak Badakhshan	Badakhshan	B	70.905	36.6831	1750	1979	○	○
672	KU11334B	North of Puli-zeribon Shewa Badakhshan	Badakhshan	B	70.488281	33.266824	-	1979	○	○
675	KU11336A	North of Puli-zeribon Shewa Badakhshan	Badakhshan	B	70.488281	33.266824	-	1979	○	○
677	KU11337	Chapchi Magzar Barak Badakhshan	Badakhshan	B	68.1261111	34.5766667	1460	1979	○	-
678	KU11338	Chapchi Magzar Barak Badakhshan	Badakhshan	B	68.1261111	34.5766667	1460	1979	○	-
679	KU11339	Chapchi Magzar Barak Badakhshan	Badakhshan	B	68.1261111	34.5766667	1460	1979	○	-
680	KU11340	Chapchi Magzar Barak Badakhshan	Badakhshan	B	68.1261111	34.5766667	1460	1979	○	○
683	KU11347	Bandi Amir	Bamyan	E	67.2147222	34.8436111	-	1979	○	○
684	KU3045	Suburbs of Kandahar	Kandahar	H	65.7615	31.6031	-	1956	○	○
685	KU3046	Suburbs of Kandahar	Kandahar	H	65.7615	31.6031	-	1956	○	○
686	KU3047	Kandahar - Jaldak	Kandahar	H	65.7615	31.6031	-	1956	○	○
688	KU3050	Kandahar - Jaldak	Kandahar	H	65.7615	31.6031	-	1956	○	○
689	KU3051	Kandahar - Jaldak	Kandahar	H	65.7615	31.6031	-	1956	-	○
690	KU3053	Kandahar - Jaldak	Kandahar	H	65.7615	31.6031	-	1956	○	○
691	KU3054	Jaldak	Zabul	H	66.7333	31.9735	-	1956	○	-
693	KU3057	Jaldak - Ghazni	Ghazni	D	66.7333	31.9735	-	1956	○	○
694	KU3059	Jaldak - Ghazni	Ghazni	D	66.7333	31.9735	-	1956	○	-

695	KU3060	Jaldak - Ghazni	Ghazni	D	66.7333	31.9735	-	1956	○	○
696	KU3062	Kabul	Kabul	F	69.168835	34.528141	-	1956	○	-
697	KU3064	Kabul	Kabul	F	69.168835	34.528141	-	1956	○	○
700	KU3068	Kabul	Kabul	F	69.168835	34.528141	-	1956	○	○
701	KU3069	Kabul	Kabul	F	69.168835	34.528141	-	1956	○	○
703	KU3071	Kabul	Kabul	F	69.168835	34.528141	-	1956	○	-
704	KU3074	Kabul	Kabul	F	69.168835	34.528141	-	1956	○	-
705	KU3076	Kabul	Kabul	F	69.168835	34.528141	-	1956	○	○
706	KU3077	Kabul	Kabul	F	69.168835	34.528141	-	1956	○	○
707	KU3078	unknown	unknown	unknown	69.171181	34.532751	-	1956	○	○
708	KU3079	unknown	unknown	unknown	69.171181	34.532751	-	1956	○	-
710	KU3081	Kabul - Doshi	Kabul	F	69.168835	34.528141	-	1956	○	○
711	KU3082	Kabul - Doshi	Kabul	F	69.168835	34.528141	-	1956	○	○
712	KU3083	17 km N of Doshi (Doshi - Pul-i-Khumri)	Baghlan	B	68.732758	35.640111	-	1956	○	-
713	KU3084	12 km S of Pul-i-Khumri(Doshi - Pul-i-Khumri)	Baghlan	B	68.722115	35.87197	-	1956	○	○
714	KU3085	Andkhui - Maimana	Faryabu	A	65.120373	36.925551	-	1956	○	○
715	KU3086	Andkhui - Maimana	Faryabu	A	65.120373	36.925551	-	1956	○	-
716	KU3087	Andkhui - Maimana	Faryabu	A	65.120373	36.925551	-	1956	○	○
717	KU3088	Andkhui - Maimana	Faryabu	A	65.120373	36.925551	-	1956	○	○
718	KU3089	Maimana	Faryabu	A	64.761991	35.930657	-	1956	○	○
719	KU3090	Maimana	Faryabu	A	64.761991	35.930657	-	1956	○	○
720	KU3091	Maimana	Faryabu	A	64.761991	35.930657	-	1956	○	○
721	KU3092	Maimana	Faryabu	A	64.761991	35.930657	-	1956	○	○
723	KU7430	Palpini Herat	Herat	C	62.202988	34.369851	1210	1967	○	○
724	KU7432	1.5 km E of Obeh (1.7 km N of Hari rud River) Herat	Herat	C	65.257873	32.274593	1360	1967	○	-
725	KU7434A	10 km E of Karokh NE of Herat Herat	Herat	C	62.579269	34.541971	1390	1967	○	-
727	KU7435	10 km E of Karokh NE of Herat Herat	Herat	C	62.579269	34.541971	1390	1967	○	○
729	KU7437	1 km E of Armalik Herat	Herat	C	69.171181	34.532751	1490	1967	○	○
730	KU7438	1 km E of Armalik Herat	Herat	C	69.171181	34.532751	1490	1967	○	-
731	KU7441	4 km E of Armalik Herat	Herat	C	62.190113	34.376681	1550	1967	○	○
733	KU7443	18 km E of Armalik Herat	Herat	C	62.190113	34.376681	1670	1967	○	○
734	KU7444	18 km E of Armalik Herat	Herat	C	62.190113	34.376681	1670	1967	○	○
735	KU7445	18 km E of Armalik Herat	Herat	C	62.190113	34.376681	1670	1967	○	○
736	KU7446A	18 km E of Armalik Herat	Herat	C	62.190113	34.376681	1670	1967	○	○
737	KU7446B	18 km E of Armalik Herat	Herat	C	62.190113	34.376681	1670	1967	○	○
739	KU7448	20 km E of Armalik Herat	Herat	C	62.190113	34.376681	1730	1967	○	○
740	KU7449	20 km E of Armalik Herat	Herat	C	62.190113	34.376681	1730	1967	○	○
744	KU7453	34 km E of Armalik Herat	Herat	C	62.190113	34.376681	1970	1967	○	○
745	KU7455	46 km E of Armalik Herat	Herat	C	62.190113	34.376681	2270	1967	○	○
746	KU7456	63 km E of Armalik Herat	Herat	C	62.190113	34.376681	1640	1967	○	○
747	KU7457	63 km E of Armalik Herat	Herat	C	62.190113	34.376681	1640	1967	○	○
748	KU7458	7 km N of Chekao Badghis	Badghis	C	63.070393	34.67989	1270	1967	○	○
749	KU7459	1.5 km W of Qala Nau Badghis	Badghis	C	63.12973	34.990179	910	1967	○	○
750	KU7460	1.5 km W of Qala Nau Badghis	Badghis	C	63.12973	34.990179	910	1967	○	○

751	KU7461	6.6 km SE of Qala Nau Badghis	Badghis	C	63.12973	34.990179	970	1967	○	○
752	KU7462	18 km SE of Qala Nau Badghis	Badghis	C	63.12973	34.990179	1060	1967	○	○
753	KU7463	35 km SE of Qala Nau Badghis	Badghis	C	63.12973	34.990179	1180	1967	○	○
754	KU7464	35 km SE of Qala Nau Badghis	Badghis	C	63.12973	34.990179	1180	1967	○	○
755	KU7465	35 km SE of Qala Nau Badghis	Badghis	C	63.12973	34.990179	1180	1967	○	○
756	KU7466	Qaddis SE of Qala Nau Badghis	Badghis	C	63.396206	35.070553	1240	1967	○	○
758	KU7468	16 km SE of Yakhak (SE of Qala Nau) Badghis	Badghis	C	63.264313	34.609023	2240	1967	○	○
759	KU7469	16 km SE of Yakhak (SE of Qala Nau) Badghis	Badghis	C	63.264313	34.609023	2240	1967	○	○
760	KU7470	Qaddis (SE of Qala Nau) Badghis	Badghis	C	63.396206	35.070553	1240	1967	○	○
761	KU7471	18 km SE of Yakhak (SE of Qala Nau) Badghis	Badghis	C	63.264313	34.609023	2180	1967	○	-
762	KU7472	20 km SE of Yakhak (SE of Qala Nau) Badghis	Badghis	C	63.264313	34.609023	2150	1967	○	-
764	KU7475	20 km SE of Yakhak (SE of Qala Nau) Badghis	Badghis	C	63.264313	34.609023	2150	1967	○	-
765	KU7476	3 km W of Karakh (NE of Herat) Herat	Herat	C	66.038361	32.331934	1300	1967	○	-
766	KU7479	66 km S of Herat Herat	Herat	C	62.082825	34.285815	1490	1967	○	○
767	KU7480	Junction of Farah rud and Khorbordar River Ghor	Ghor	C	64.845886	34.113169	910	1967	○	○
768	KU7481	Junction of Farah rud and Khorbordar River Ghor	Ghor	C	64.845886	34.113169	910	1967	○	-
769	KU7482	Junction of Farah rud and Khorbordar River Ghor	Ghor	C	64.845886	34.113169	910	1967	○	○
770	KU7483	108 km S of Herat Herat	Herat	C	62.082825	34.285815	1120	1967	○	-
771	KU7484	63 km S of Herat Herat	Herat	C	62.082825	34.285815	1360	1967	○	○
773	KU7487	13 km N of Obeh Herat	Herat	C	65.257873	32.274593	1420	1967	○	○
774	KU7489	36 km W of Besha Herat	Herat	C	69.153385	34.540868	1360	1967	○	-
776	KU7491	26 km W of Besha Herat	Herat	C	69.153385	34.540868	1420	1967	○	-
777	KU7493	13 km W of Besha Herat	Herat	C	69.153385	34.540868	1520	1967	○	○
779	KU7495	13 km W of Besha Herat	Herat	C	69.153385	34.540868	1520	1967	○	○
780	KU7496	1.6 km S of Dakhan Herat	Herat	C	63.434601	33.950879	1550	1967	○	○
781	KU7497	1.6 km S of Dakhan Herat	Herat	C	63.434601	33.950879	1550	1967	○	○
782	KU7498	1.6 km S of Dakhan Herat	Herat	C	63.434601	33.950879	1550	1967	○	-
783	KU7499	1.6 km S of Dakhan Herat	Herat	C	63.434601	33.950879	1550	1967	○	○
784	KU7502	3 km SE of Margha Ghor	Ghor	C	64.254885	34.347872	1880	1967	○	-
785	KU7503	3 km SE of Margha Ghor	Ghor	C	64.254885	34.347872	1880	1967	○	○
786	KU7504	3 km SE of Margha Ghor	Ghor	C	64.254885	34.347872	1880	1967	○	-
787	KU7505	5 km SE of Margha Ghor	Ghor	C	64.254885	34.347872	2120	1967	○	○
788	KU7506	16 km SE of Margha Ghor	Ghor	C	64.254885	34.347872	2240	1967	○	○
790	KU7508	16 km SE of Margha Ghor	Ghor	C	64.254885	34.347872	2240	1967	○	○
791	KU7509	16 km SE of Margha Ghor	Ghor	C	64.254885	34.347872	2240	1967	○	○
792	KU7510	16 km SE of Margha Ghor	Ghor	C	64.254885	34.347872	2240	1967	○	○
794	KU7512	10 km NW of Targi-Azao Ghor	Ghor	C	64.914379	34.100235	2490	1967	○	○
795	KU7513	10 km NW of Targi-Azao Ghor	Ghor	C	64.914379	34.100235	2490	1967	○	○
796	KU7514	10 km NW of Targi-Azao Ghor	Ghor	C	64.914379	34.100235	2490	1967	○	○
797	KU7515	15 km NW of Targi-Azao Ghor	Ghor	C	64.914379	34.100235	2490	1967	○	○
798	KU7516	Tagao Ishan River valley 3 km E of Qala Hissar W of Qala Shararak Ghor	Ghor	C	64.048805	34.163125	2180	1967	○	○
804	KU7522	3 km S of Tagao Ishan valley E of Qala Hissar W of Qala Shararak Ghor	Ghor	C	64.048805	34.163125	2300	1967	○	○
806	KU7524	3 km E of Qala Hissar (W of Qala Shararak) on Tagao Ishan River Ghor	Ghor	C	64.048805	34.163125	2180	1967	○	○
810	KU7528	83 km SW of Djam near Qala Shararak Ghor	Ghor	C	64.225702	34.119948	2390	1967	○	○

812	KU7530	83 km SW of Djam near Qala Shararak Ghor	Ghor	C	64.225702	34.119948	2390	1967	○	○
813	KU7531	3 km S of Gok Tago near Shararak Ghor	Ghor	C	64.239607	34.122648	2300	1967	○	○
815	KU7533	6 km E of Qala Shararak Ghor	Ghor	C	64.225702	34.119948	2390	1967	○	○
816	KU7534	6 km E of Qala Shararak Ghor	Ghor	C	64.225702	34.119948	2390	1967	○	○
817	KU7535	6 km E of Qala Shararak Ghor	Ghor	C	64.225702	34.119948	2390	1967	○	-
819	KU7537	6 km E of Qala Shararak Ghor	Ghor	C	64.225702	34.119948	2390	1967	○	-
821	KU7539	11 km E of Qala Shararak Ghor	Ghor	C	64.225702	34.119948	2490	1967	○	○
822	KU7540	11 km E of Qala Shararak Ghor	Ghor	C	64.225702	34.119948	2490	1967	○	○
823	KU7542	16 km E of Qala Shararak Ghor	Ghor	C	64.225702	34.119948	2490	1967	○	○
824	KU7547	15 km SE of Dosht-i-Pam (on Tairama road) Ghor	Ghor	C	64.904251	34.081441	2640	1967	○	○
826	KU7551	36 km SE of Dosht-i-Pam (on Tairama road) Ghor	Ghor	C	64.904251	34.081441	2490	1967	○	○
827	KU7553	36 km SE of Dosht-i-Pam (on Tairama road) Ghor	Ghor	C	64.904251	34.081441	2490	1967	○	○
828	KU7555	36 km SE of Dosht-i-Pam (on Tairama road) Ghor	Ghor	C	64.904251	34.081441	2490	1967	○	○
829	KU7556	36 km SE of Dosht-i-Pam (on Tairama road) Ghor	Ghor	C	64.904251	34.081441	2490	1967	○	○
831	KU7558	60 km SE of Dosht-i-Pam (on Tairama road) Ghor	Ghor	C	64.904251	34.081441	2610	1967	○	○
832	KU7559	60 km SE of Dosht-i-Pam (on Tairama road) Ghor	Ghor	C	64.904251	34.081441	2610	1967	○	-
833	KU7560	60 km SE of Dosht-i-Pam (on Tairama road) Ghor	Ghor	C	64.904251	34.081441	2610	1967	○	○
835	KU7562	Tarbulock 58 km NE of Qala Shararak Ghor	Ghor	C	64.225702	34.119948	2640	1967	○	○
839	KU7566	23 km E of Dosht-i-Pam Ghor	Ghor	C	64.904251	34.081441	2640	1967	○	○
841	KU7568	35 km E of Dosht-i-Pam Ghor	Ghor	C	64.904251	34.081441	2850	1967	○	○
842	KU7569	35 km E of Dosht-i-Pam Ghor	Ghor	C	64.904251	34.081441	2850	1967	○	○
843	KU7570	35 km E of Dosht-i-Pam Ghor	Ghor	C	64.904251	34.081441	2850	1967	○	○
845	KU7572	46 km NE of Dosht-i-Pam Ghor	Ghor	C	64.904251	34.081441	2640	1967	○	○
847	KU7574	46 km NE of Dosht-i-Pam Ghor	Ghor	C	64.904251	34.081441	2640	1967	○	○
848	KU7575	46 km NE of Dosht-i-Pam Ghor	Ghor	C	64.904251	34.081441	2640	1967	○	○
849	KU7576	3 km W of Qala Ahangharan Ghor	Ghor	C	65.126037	34.322116	2120	1967	○	○
850	KU7577	3 km W of Qala Ahangharan Ghor	Ghor	C	65.126037	34.322116	2120	1967	○	○
851	KU7578	3 km W of Qala Ahangharan Ghor	Ghor	C	65.126037	34.322116	2120	1967	○	○
854	KU7582	11 km W of Qala Ahangharan Ghor	Ghor	C	65.126037	34.322116	2180	1967	○	○
857	KU7585	1.6 km W of Qala Ahangharan Ghor	Ghor	C	65.126037	34.322116	2240	1967	○	○
858	KU7586	1.6 km W of Qala Ahangharan Ghor	Ghor	C	65.126037	34.322116	2240	1967	○	-
859	KU7587	11 km S of Kilmin Ghor	Ghor	C	64.92897	34.10646	2580	1967	○	○
862	KU7592	Kilmin Ghor	Ghor	C	64.92897	34.10646	2090	1967	○	-
863	KU7593	Kilmin Ghor	Ghor	C	64.92897	34.10646	2090	1967	○	○
864	KU7595	Kilmin Ghor	Ghor	C	64.92897	34.10646	2090	1967	○	○
865	KU7596	20 km E of Qala Ahangharan Ghor	Ghor	C	65.126037	34.322116	2300	1967	○	-
866	KU7597	20 km E of Qala Ahangharan Ghor	Ghor	C	65.126037	34.322116	2300	1967	○	○
867	KU7598	15 km E of Badgah Ghor	Ghor	C	65.424342	34.514924	2420	1967	○	-
869	KU7600	15 km E of Badgah Ghor	Ghor	C	65.424342	34.514924	2420	1967	○	-
870	KU7602	Daulat Yar Ghor	Ghor	C	64.845886	34.113169	2460	1967	○	○
871	KU7604	40 km N of Herat Herat	Herat	C	62.082825	34.285815	1490	1967	○	○
872	KU7605	Gormos village Ghor	Ghor	C	64.845886	34.113169	2760	1967	○	○
873	KU7606	15 km E of Qizil Ghor	Ghor	C	66.020164	34.459183	2880	1967	○	-
875	KU7608	30 km E of Qizil Ghor	Ghor	C	66.020164	34.459183	2790	1967	○	○

876	KU7609	3 km E of Qala Sakawa (Daulat Yar - Panjao) Ghor	Ghor	C	66.274717	34.498751	2790	1967	○	○
877	KU7610	3 km E of Qala Sakawa (Daulat Yar - Panjao) Ghor	Ghor	C	66.274717	34.498751	2790	1967	-	○
878	KU7611	3 km E of Qala Sakawa (Daulat Yar - Panjao) Ghor	Ghor	C	66.274717	34.498751	2790	1967	○	○
879	KU7612	3 km E of Qala Sakawa (Daulat Yar - Panjao) Ghor	Ghor	C	66.274717	34.498751	2790	1967	○	○
880	KU7613	33 km E of Qala Sakawa (Daulat Yar - Panjao) Ghor	Ghor	C	66.274717	34.498751	3000	1967	○	○
881	KU7614	5 km W of Banki (Daulat Yar - Panjao) Ghor	Ghor	C	64.402428	33.45224	2910	1967	○	-
882	KU7615	5 km W of Banki (Daulat Yar - Panjao) Ghor	Ghor	C	64.402428	33.45224	2910	1967	○	-
883	KU7616	15 km W of Panjao Bamiyan	Bamyan	E	67.006874	34.417843	2910	1967	○	○
884	KU7617	21 km N of Panjao Bamiyan	Bamyan	E	67.006874	34.417843	3030	1967	○	○
885	KU7618	6.6 km N of Panjao Bamiyan	Bamyan	E	67.006874	34.417843	2790	1967	○	○
886	KU7619	3 km S of Naik (Panjao - Bamian) Bamiyan	Bamyan	E	67.00676	34.744208	2730	1967	○	○
887	KU7620	11 km E of Naik (Panjao - Bamian) Bamiyan	Bamyan	E	67.00676	34.744208	2700	1967	○	○
888	KU7621	11 km E of Naik (Panjao - Bamian) Bamiyan	Bamyan	E	67.00676	34.744208	2700	1967	○	○
889	KU7622	16 km E of Naik (Panjao - Bamian) Bamiyan	Bamyan	E	67.00676	34.744208	2940	1967	○	○
890	KU7623	Shinbatu (Panjao - Bamian) Bamiyan	Bamyan	E	68.021393	34.940661	3090	1967	○	○
891	KU7624	15 km W of Bamian (Panjao - Bamian) Bamiyan	Bamyan	E	67.790909	34.841197	2640	1967	○	○
892	KU7626	Bamian (Panjao - Bamian) Bamiyan	Bamyan	E	67.790909	34.841197	2580	1967	○	○
893	KU7627	6.6 km E of Bamian Bamiyan	Bamyan	E	67.790909	34.841197	2520	1967	○	-
896	KU7634	near Shutue Shahe village Parwan	Parwan	E	68.957977	35.185706	1820	1967	○	-
897	KU7637	5 km E of Unai Pass Wardak	Wardak	F	68.376102	34.450605	2850	1967	○	-
898	KU7638	5 km E of Unai Pass Wardak	Wardak	F	68.376102	34.450605	2850	1967	○	-
899	KU7640	Unai Pass - below peak Wardak	Wardak	F	68.376102	34.450605	3000	1967	○	○
901	KU7642	Unai (below Pass) Wardak	Wardak	F	68.376102	34.450605	3000	1967	○	○
903	KU7644	Taliqan Takar	Takhar	B	69.166259	34.566288	850	1967	○	○
904	KU7646	5 km W of Mazar-i-Sharif Balkh	Balkh	A	67.109613	36.703288	420	1967	○	○
905	KU7647	5 km W of Mazar-i-Sharif Balkh	Balkh	A	67.109613	36.703288	420	1967	○	-
906	KU7648	25 km S of Tash-Kurghan near Khulm Samangan	Samangan	A	67.693105	36.687026	790	1967	○	○
907	KU7649	3 km S of Daulatabad Balkh	Balkh	A	66.814982	36.98874	390	1967	○	○
909	KU7651	3 km S of Daulatabad Balkh	Balkh	A	66.814982	36.98874	390	1967	○	○
910	KU7652	3 km S of Daulatabad Balkh	Balkh	A	66.814982	36.98874	390	1967	○	○
915	KU7657	25 km S of Tash-Kurghan near Khulm Samangan	Samangan	A	67.693105	36.687026	790	1967	○	○
916	KU7658	5 km SW of Haibak Samangan	Samangan	A	68.026886	36.240507	1030	1967	○	○
921	KU7663	8 km S of Salang Pass (Doshi - Charikar) Baghlan	Baghlan	B	68.689041	35.837076	2670	1967	○	○
922	KU7664	43 km SW of Ghazni Ghazni	Ghazni	D	68.378448	33.535214	2090	1967	○	-
923	KU7665	43 km SW of Ghazni Ghazni	Ghazni	D	68.378448	33.535214	2090	1967	○	○
924	KU7666	105 km SW of Ghazni Ghazni	Ghazni	D	68.378448	33.535214	2070	1967	○	○
925	KU7667	93 km NE of Kandahar Kandahar	Kandahar	H	65.756207	31.599387	1390	1967	○	○
926	KU7668	45 km NE of Kandahar Kandahar	Kandahar	H	65.756207	31.599387	1210	1967	○	○
927	KU7669	16 km W of Kandahar Kandahar	Kandahar	H	65.756207	31.599387	1000	1967	○	○
929	KU7671	75 km W of Kandahar Kandahar	Kandahar	H	65.756207	31.599387	1000	1967	○	○
931	KU7673	-	unknown	unknown	69.171181	34.532751	-	1967	○	○
932	KU7675	-	unknown	unknown	69.171181	34.532751	-	1967	○	○
933	KU7676	-	unknown	unknown	69.171181	34.532751	-	1967	○	○
934	KU7678	-	unknown	unknown	69.171181	34.532751	-	1967	○	○

935	KU7679	-	unknown	unknown	69.171181	34.532751	-	1967	○	○
936	KU7681	-	unknown	unknown	69.171181	34.532751	-	1967	○	○
939	KU7683	-	unknown	unknown	69.171181	34.532751	-	1967	○	○
940	KU7684	-	unknown	unknown	69.171181	34.532751	-	1967	○	-
941	KU7686	-	unknown	unknown	69.171181	34.532751	-	1967	○	-
942	KU7689	-	unknown	unknown	69.171181	34.532751	-	1967	○	-
943	KU7690	-	unknown	unknown	69.171181	34.532751	-	1967	○	-
Check1	Herat 99	-	-	-	-	-	-	-	○	○
Check2	Mazar 99	-	-	-	-	-	-	-	○	○
Check3	Kabul 2000	-	-	-	-	-	-	-	○	○
Check4	Sulh 02	-	-	-	-	-	-	-	○	○
Check5	Bakhtawar	-	-	-	-	-	-	-	○	○
Check6	Lalmi 2	-	-	-	-	-	-	-	○	○
Check7	Rana 96	-	-	-	-	-	-	-	○	○

[a] Variety names are shown for seven check varieties

[b] National Bio-Resource Project, Japan
